# High Conductivity, Semiconducting, and Metallic PEDOT:PSS Electrode for All-Plastic Solar Cells

**DOI:** 10.3390/molecules28062836

**Published:** 2023-03-21

**Authors:** Shisong Nie, Fei Qin, Yanfeng Liu, Chufeng Qiu, Yingzhi Jin, Hongmei Wang, Lichun Liu, Lin Hu, Zhen Su, Jiaxing Song, Xinxing Yin, Zhiguang Xu, Yuyuan Yao, Hao Wang, Yinhua Zhou, Zaifang Li

**Affiliations:** 1 National Engineering Lab of Textile Fiber Materials & Processing Technology (Zhejiang), Zhejiang Sci-Tech University, Hangzhou 310018, China; 2 China-Australia Institute for Advanced Materials and Manufacturing (IAMM), Jiaxing University, Jiaxing 314001, China; 3 Wuhan National Laboratory for Optoelectronics and School of Optical and Electronic Information, Huazhong University of Science and Technology, Wuhan 430074, China; 4 Department of Physics, Chemistry and Biology, Linköping University, SE-58183 Linköping, Sweden; 5 College of Biological, Chemical Sciences and Engineering, Jiaxing University, Jiaxing 314001, China

**Keywords:** PEDOT:PSS, flexible electrodes, high conductivity, all-plastic solar cells

## Abstract

Plastic electrodes are desirable for the rapid development of flexible organic electronics. In this article, a plastic electrode has been prepared by employing traditional conducting polymer poly(3,4-ethylenedioxythiophene):poly(styrene sulfonate) (PEDOT:PSS) and plastic substrate polyethersulfone (PES). The completed electrode (Denote as HC-PEDOT:PSS) treated by 80% concentrated sulfuric acid (H_2_SO_4_) possesses a high electrical conductivity of over 2673 S/cm and a high transmittance of over 90% at 550 nm. The high conductivity is attributed to the regular arrangement of PEDOT molecules, which has been proved by the X-ray diffraction characterization. Temperature-dependent conductivity measurement reveals that the HC-PEDOT:PSS possesses both semiconducting and metallic properties. The binding force and effects between the PEDOT and PEI are investigated in detail. All plastic solar cells with a classical device structure of PES/HC-PEDOT:PSS/PEI/P3HT:ICBA/EG-PEDOT:PSS show a PCE of 4.05%. The ITO-free device with a structure of Glass/HC-PEDOT:PSS/Al4083/PM6:Y6/PDINO/Ag delivers an open-circuit voltage (*V*_OC_) of 0.81 V, short-circuit current (*J*_SC_ ) of 23.5 mA/cm^2^, fill factor (FF) of 0.67 and a moderate power conversion efficiency (PCE) of 12.8%. The above results demonstrate the HC-PEDOT:PSS electrode is a promising candidate for all-plastic solar cells and ITO-free organic solar cells.

## 1. Introduction

Organic solar cells (OSCs) have attracted dramatically increasing attention due to their advantages of low cost, easy fabrication, light weight, and good flexibility. An over 19% power conversion efficiency (PCE) of OSC has been reported recently [1,2], demonstrating its promising future for application prospects. However, most of these OSCs are fabricated based on indium tin oxide (ITO) and vacuum-deposited metals as bottom and top electrodes, respectively. Although these materials possess high electrical conductivity, the corresponding devices demonstrate poor flexibility, high cost, and complicated processing procedures. Therefore, plastic electrodes, with advantages including high flexibility and conductivity, low cost, and good compatibility with solution processing, are desired for constructing all-plastic solar cells.

The conducting polymer poly(3,4-ethylenedioxythiophene):poly(styrenesulfonate) (PEDOT:PSS) has been considered to be one of the most promising candidates as electrode materials, owing to its advantages of aqueous solution processable, excellent stability, high transparency over 90%, and high conductivity over 10^3^ S/cm [3,4,5,6]. The electrical conductivity of PEDOT:PSS is strongly dependent on the ratio of PEDOT and PSS, PEDOT arrangement, and film morphology, which can be strongly modified via second doping or post-treatments to the film. It has been reported that the electrical conductivity of PEDOT:PSS can be enhanced over two or three orders of magnitudes by doping polar organic solvents such as dimethylformamide (DMF), dimethyl sulfoxide (DMSO), ethylene glycol (EG), glycerol and sorbitol [7,8,9,10]. Surfactant is another effective dopant for boosting both the electrical conductivity and the wetting property of PEDOT:PSS films [11]. Ionic liquids are also proven to be an operative dopant that can bring electrical conductivity from 1 S/cm for pristine PEDOT:PSS to 1280 S/cm for the sample with 1.5 wt% 1-ethyl-3-methylimidazolium tetracyanoborate [12]. Moreover, a transmittance of 91.5% at 550 nm was achieved. Post-treatments of the films are another method to improve the conductivity of PEDOT:PSS. Polar organic solvents, aqueous or organics solutions of salts, and different acids are all proven to be effective for this purpose [13,14,15,16]. Among them, concentrated H_2_SO_4_ is the most effective method to enhance the electrical conductivity of PEDOT:PSS films. Ouyang et al. first reported a very high conductivity of 3065 S/cm for H_2_SO_4_ post-treated PEDOT:PSS films, whereas, the pristine PEDOT:PSS film only has a conductivity of 0.3 S/cm [17]. The transmittance of the H_2_SO_4_ post-treated PEDOT:PSS film (66 nm) is 87% at 550 nm. Lee’s group further boosted the electrical conductivity from 1 S/cm to 4380 S/cm by optimizing the post-treated conditions with H_2_SO_4_ [13]. The obtained film has a transmittance of 90% at 550 nm. Up to now, the highest electrical conductivity of PEDOT:PSS treated by H_2_SO_4_ has reached 4840 S/cm, a PEDOT:PSS with 788 S/cm conductivity as the reference. PEDOT:PSS films treated by H_2_SO_4_ possess unique metallic properties, indicating the regular arrangement of the PEDOT chains [18].

In 2002, Zhang et al. first demonstrated that sorbitol-doped PEDOT:PSS could be used as transparent electrodes in OSCs due to its moderate sheet resistance of 1000 ohm/sq and an appropriate work function [19]. OSCs with a structure of Glass/S-PEDOT:PSS/MEH-PPV/PCBM/Al demonstrated an open-circuit voltage (*V_OC_*) of 0.75 V, short-circuit current (*J_SC_*) of 1.6 mA/cm^2^, fill factor (FF) of 0.24 and a PCE of 0.36%. After that, more researchers carried out the research of PEDOT:PSS based electrodes and their applications on OSCs and other types of solar cells [20,21,22]. With the development of PEDOT:PSS-based flexible electrodes, all plastic solar cells with PEDOT:PSS electrodes as bottom and top electrodes have aroused people’s interests. In 2009, Hau et al. demonstrated the first all-plastic solar cell using DMSO-doped PEDOT:PSS as the bottom cathode and the top anode. With a device structure of PEDOT:PSS-DMSO/ZnO/C60-SAM/P3HT:PCBM/PEDOT:PSS/PEDOT:PSS-DMSO, the optimized device delivered a *V_OC_* of 0.31 V, *J_SC_* of 5.49 mA/cm^2^, FF of 0.28, and a low PCE of 0.47% [23]. Zhou et al. developed a universal method to adjust the work function of different electrodes with PEI. The all-plastic solar cells achieved an average PCE of 3.5%, with PEI-coated PH1000 as the bottom electrode, and high conductivity PEDOT:PSS as the top electrode [24]. The same group also reported on the first demonstration of semi-transparent all-plastic solar cells fabricated in ambient air by sequential dry film-transfer lamination of a P3HT:ICBA photoactive layer and a PEDOT:PSS top electrode. This laminated all plastic devices displayed a *V_OC_* of 0.80 V, *J_SC_* of 5.6 mA/cm^2^, FF of 0.55, and a PCE of 2.4% [25]. Li et al. fabricated a PEDOT:PSS electrode (low-WF PEDOT:PSS) with high conductivity of 1561 S/cm and low work function of 4.0 eV, by a two-step dipping process in H_2_SO_4_ and PEI solution, photovoltaic devices with a structure of glass/low-WF PEDOT:PSS/P3HT:ICBA/high WF-PEDOT:PSS showed a *V_OC_* of 0.81 V, *J_SC_* of 8.1 mA/cm^2^, FF of 0.61, and a champion PCE of 4.0% [26]. In the same year, Li et al. found that PEI can decrease the conductivity and transparency of H_2_SO_4_ treated PEDOT:PSS films, the amount of the decline depends on the PEI’s concentration and treatment time [27]. Although H_2_SO_4_ treatment can bring high conductivity to PEDOT:PSS, its compatibility with flexible substrates such as PES, PET and PFN is poor. None of the above flexible substrates can resist corrosion from H_2_SO_4_. To avoid the corrosion of plastic substrates, Meng et al. treated PEDOT:PSS electrodes by employing phosphoric acid (H_3_PO_4_) and obtained a high conductivity of 1460 S/cm [28]. Using PES/H_3_PO_4_-treated PEDOT:PSS flexible electrode as the bottom electrode and P3HT:ICBA as the active layer, a *V_OC_* of 0.84 V, *J_SC_* of 6.6 mA/cm^2^, FF of 0.60, and a PCE of 3.3% was obtained. Koppitz et al. reported an all-plastic OSC with PEDOT:PSS as the top and bottom electrodes embedded in AgNWs [29]. The doctor-blading device manufactured in air showed good robustness in bending experiments, a PCE of 3.8% was achieved with PBTZT-stat-BDTT-8:PCBM as the active layer. Although the transfer laminated processing can transfer sulfuric acid-treated PEDOT:PSS films from glass to flexible substrates, highly precise tuning of surface energy and interface adhesion are required. H_3_PO_4_ post-treated processing can solve this problem, which can bring a flexible transparent PEDOT:PSS electrode with a reasonable electrical conductivity over 1400 S/cm on PES substrate [28]. However, this electrical conductivity is still much lower than that of H_2_SO_4_ post-treatment. We carried out some works on H_2_SO_4_ treated films, especially the free-standing PEDOT:PSS films, which have been applied onto supercapacitors and thermoelectric devices, thanks to their high electrical conductivity of over 2500 S/cm [30,31]. However, the compatibility of different concentrations of sulfuric acid treatment on flexible substrates, and the electrical conductivity of the resulting PEDOT:PSS films have not been discussed in detail.

Herein, a plastic electrode (denote as HC-PEDOT:PSS) was prepared by employing PEDOT:PSS and plastic substrate polyethersulfone (PES). This electrode treated by H_2_SO_4_ possesses high electrical conductivity of 2673 S/cm and high transmittance of over 90% at 550 nm. The outstanding conductivity is mainly attributed to the regular arrangement of PEDOT molecules, which is proved by the X-ray diffraction (XRD) characterizations. Both semiconducting and metallic properties have been observed by temperature-dependent conductivity measurement. The binding force and effects between the PEDOT and PEI are investigated in detail, demonstrating a strong Coulomb force between these two molecules. The surface morphology of pristine PEDOT:PSS and HC-PEDOT:PSS films are investigated by atomic force microscopy (AFM) and transmission electron microscope (TEM), from which we find that both of the above films possess very smooth surfaces. All plastic solar cells with a device structure of PES/HC-PEDOT:PSS/PEI/P3HT:ICBA/EG-PEDOT:PSS are fabricated. A *V_OC_* of 0.79 V, *J_SC_* of 8.99 mA/cm^2^, FF of 0.57, with a decent PCE of 4.05% has been achieved, this is the highest performance among reported single junction all plastic OSCs. The ITO-free device with a structure of Glass/HC-PEDOT:PSS/Al4083/PM6:Y6/PDINO/Ag was also fabricated by employing PM6:Y6 as the active layer, showing a *V_OC_* of 0.81 V, *J_SC_* of 23.5 mA/cm^2^, FF of 0.67, and a moderate PCE of 12.8%. Our research demonstrates the HC-PEDOT:PSS electrode is a promising candidate for all-plastic solar cells and ITO-free OSCs.

## 2. Results and Discussion

**Figure 1:**
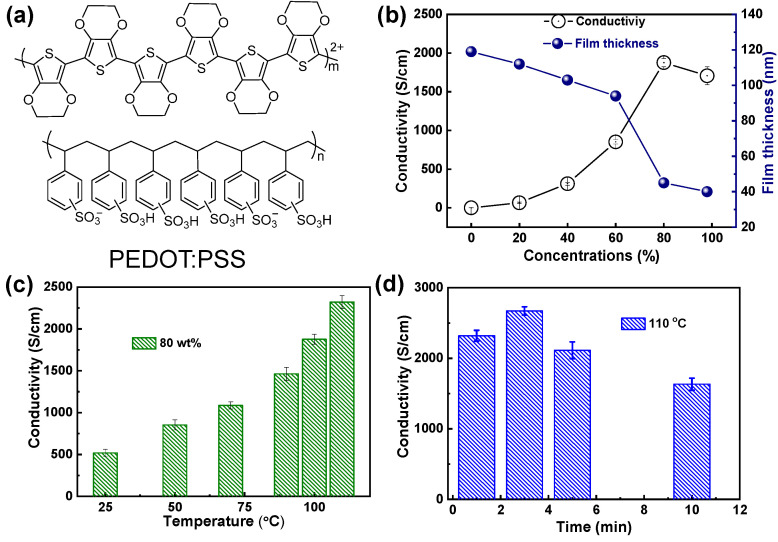
(**a**) Molecular structures of PEDOT and PSS, (**b**) The conductivity variation of PEDOT:PSS films treated with different concentrations H_2_SO_4_ (0 wt%, 20 wt%, 40 wt%, 60 wt%, 80 wt%, 98 wt%), (**c**) Temperature-dependent conductivity of PEDOT:PSS films treated with 80 wt% H_2_SO_4_ for 1 min, (**d**) Conductivity optimization of PEDOT:PSS films treated with 80 wt% H_2_SO_4_ under different treating time.

The molecular structures of PEDOT and PSS are shown in Figure 1a. PEDOT exhibits the positive charge and PSS possesses the negative charge, thus these two molecules are tightly bound by coulomb forces. As for the PSS, it is mainly used to coat insoluble PEDOT molecules to form nano-size particles and then disperse them into the aqueous solution for easy processing. PEDOT, on the other hand, mainly plays the role of conducting electricity in the film. Figure 1b shows the conductivity and film thickness variations of PEDOT:PSS films treated by H_2_SO_4_ with different concentrations (0 wt%, 20 wt%, 40 wt%, 60 wt%, 80 wt%, and 98 wt%) at 100 °C for 1 min. We find that the conductivity increases gradually with the decrease in film thickness. the highest conductivity is realized at a concentration of 80 wt%. This is mainly due to the removal of PSS and enhanced ordered-arrangement of PEDOT [32]. Then the temperature and time-dependent conductivity variations of PEDOT:PSS are investigated as well. As shown in Figure 1c, the conductivity increases gradually with the increase in temperature. However, the PES substrate will be damaged when the temperature exceeded 110 °C. The time intervals of the heat treatment at 110 °C are also optimized. As shown in Figure 1d, a champion conductivity of 2673 S/cm is realized under the treating time of 3 min.

**Figure 2:**
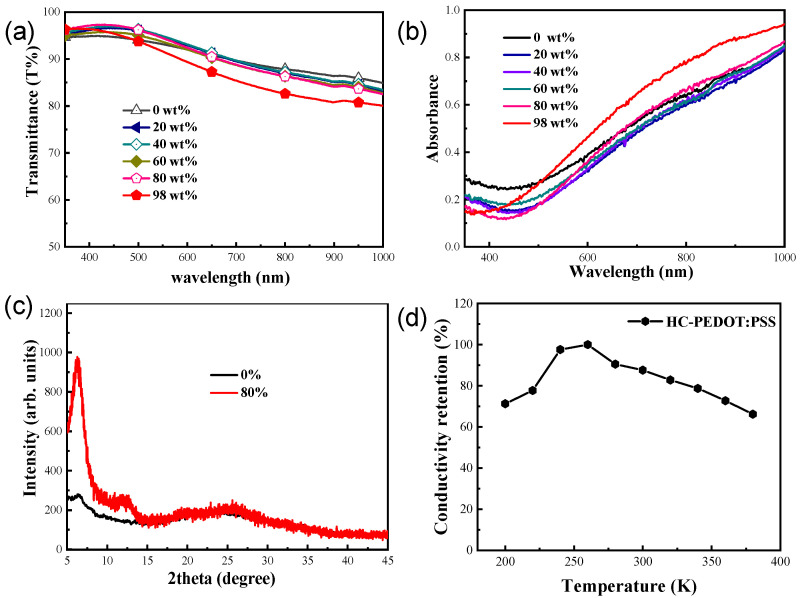
(**a**) Transmittance and (**b**) absorbance of PEDOT:PSS films treated by H_2_SO_4_ with different concentrations (0 wt%, 20 wt%, 40 wt%, 60 wt%, 80 wt%, 98 wt%), (**c**) XRD characteristics of 80 wt% H_2_SO_4_ treated PEDOT:PSS film and pristine PEDOT:PSS (PH1000) film, (**d**) the conductivity-temperature dependent test on HC-PEDOT:PSS film.

Figure 2a shows the transmittance spectra of PEDOT:PSS films on glass substrates treated by H_2_SO_4_ with different concentrations (0 wt%, 20 wt%, 40 wt%, 60 wt%, 80 wt%, and 98 wt%) at 100 °C for 1 min. These films exhibit similar and high transmittance, except for the 98 wt% concentrated sulfuric acid treated PEDOT:PSS, which shows an obvious reduction of transmittance in the visible range. This result also proves that 80 wt% sulfuric acid treatment can provide better optical performance than that of 98 wt% sulfuric acid-treated films. Figure 2b shows the absorbance spectra of the above PEDOT:PSS films, from which we can find that the 98% sulfuric acid treatment brings a significant increase in absorption, which is consistent well with the transmittance measurements. The diffused surface reflectance spectra of the above PEDOT:PSS films are shown in Figure S1 in Supporting Information, a higher reflectance could be found for the 98% sulfuric acid treated film, which is consistent well with the absorption and transmittance measurements.

In order to compare the structural changes of PEDOT:PSS (PH1000) and HC-PEDOT:PSS films, XRD measurement is performed. Figure 2c presents the XRD patterns of these two films. As for the HC-PEDOT:PSS films treated by the diluted 80 wt% sulfuric acid, the peak intensity at around 6.0° and 12° significantly increase, suggesting a better molecular stacking and arrangement of PEDOT. This result is well consistent with the increase in electrical conductivity. To investigate the relationship between the molecular arrangement of PEDOT and temperature, we conducted a conductivity-temperature-dependent test on HC-PEDOT:PSS. As shown in Figure 2d, the conductivity increases with the increase of temperature in the low temperature region from 200 to 260 K, showing the property of organic semiconductors [33]. After the temperature exceeds 260 K, the conductivity decreases with the increase in temperature, showing a metallic characteristic. This result reveals that this HC-PEDOT:PSS electrode possesses both semiconducting and metallic properties.

**Figure 3:**
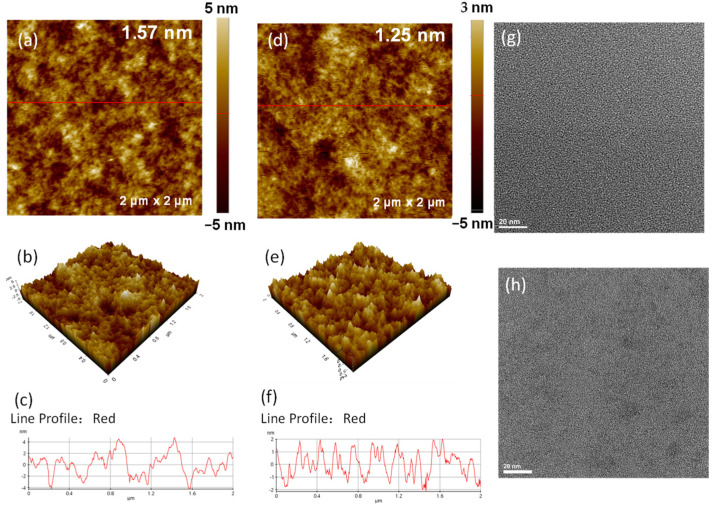
AFM height images of PEDOT:PSS films treated with 0 wt% (**a**) and 80 wt% (**d**) H_2_SO_4_. AFM 3D images of PEDOT:PSS films treated with 0 wt% (**b**) and 80 wt% (**e**) H_2_SO_4_. Line profiles PEDOT:PSS films treated with 0 wt% (**c**) and 80 wt% (**f**) H_2_SO_4_. TEM images of PEDOT:PSS films treated with 0 wt% (**g**) and 80 wt% (**h**) H_2_SO_4_.

As shown in Figure 3a–f, the morphology of pristine PEDOT:PSS and HC-PEDOT:PSS films are investigated by atomic force microscopy (AFM), from which we find that both of the above films possess very smooth surfaces. The 80 wt% H_2_SO_4_ treated PEDOT:PSS film shows a root-mean-square (RMS) value of 1.25 nm, slightly smoother than that of pristine PEDOT:PSS film (1.57 nm). Figure 3g,h shows the transmission electron microscope (TEM) images of the above two PEDOT:PSS films. The pristine PEDOT:PSS film shows a homogeneous morphology while the HC-PEDOT:PSS film demonstrates some PEDOT domains, which should be caused by the partial removal of PSS and thus a regular aggregation of PEDOT molecules. This also could be confirmed by the XRD result discussed before.

Both the conductivity and work function are key parameters for electrodes. As for the pristine PEDOT:PSS (PH1000), it shows a low conductivity of 0.98 S/cm and a high work function value 5.22 eV (Table 1). After being treated with 80 wt% H_2_SO_4_, the conductivity of this PEDOT:PSS electrode was boosted to 2673 S/cm dramatically while the work function decreased slightly to 5.12 eV, attributed to the partial removal of PSS. The work function was then further lowered to 4.22 eV after being modified by polyethylenimine (PEI). We also noticed the conductivity of the HC-PEDOT:PSS electrode was reduced to 1744 S/cm after the PEDOT reacted with PEI. In order to investigate the binding force and effects between the PEDOT and PEI, the achieved HC-PEDOT:PSS (PEI) electrode was further treated by 80 wt% H_2_SO_4_. We find that the conductivity recovers to 2294 S/cm while the work function revives to 4.53 eV. This means the PEI can be moved greatly by 80 wt% H_2_SO_4_ treatment and thus eliminate largely the PEI’s influence on PEDOT’s conductivity. We speculate there is still partial PEI residual on HC-PEDOT:PSS surface, based on its low work function value of 4.53 eV. The above research demonstrates that there exists a strong Coulomb force between PEDOT and PEI.

**Figure 4:**
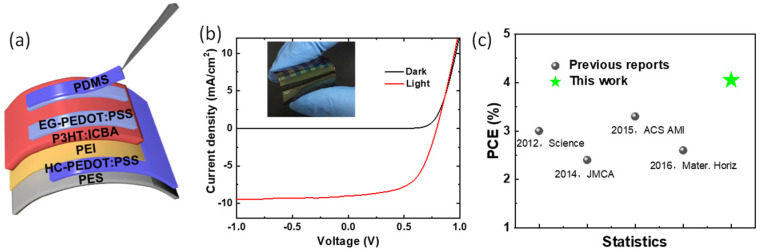
(**a**) Illustration of all plastic solar cells with a device structure of PES/HC-PEDOT:PSS/PEI/P3HT:ICBA/EG-PEDOT:PSS, (**b**) *J*-*V* curves of all plastic OSCs with HC-PEDOT:PSS as bottom electrodes, EG-PEDOT:PSS as the top electrode and P3HT:ICBA as the active layer. (**c**) PCE statistics of the single-junction all plastic solar cells reported before and this work [24,25,28,34].

Considering the high transmittance, superior conductivity, and suitable work function of PEI-modified HC-PEDOT:PSS electrode, we fabricated all-plastic OSCs with a device structure of PES/HC-PEDOT:PSS/PEI/P3HT:ICBA/EG-PEDOT:PSS (Figure 4a) by fully solution-processing technology. The inset in Figure 4b is a picture of an all-plastic solar cell with the above device structure, from which we observed the excellent flexibility of this device. The *J-V* characteristics of the above device are shown in Figure 4b. A good performance under the illumination is achieved, with a *V_OC_* of 0.79 V, *J_SC_* of 8.99 mA/cm^2^, FF of 0.57, and a PCE of 4.05%, which is higher than previous reports (Table 2). The improved device performance is mainly due to the increased *J_SC_*, deriving from both the high conductivity and high transmittance of HC-PEDOT:PSS electrode. In order to further explore the application of HC-PEDOT:PSS electrodes in OSCs, ITO-free devices with a structure of Glass/HC-PEDOT:PSS/Al4083/PM6:Y6/PDINO/Ag are fabricated by employing PM6:Y6 as the active layer. The optimized devices show a *V_OC_* of 0.81 V, *J_SC_* of 23.5 mA/cm^2^, FF of 0.67, and a moderate PCE of 12.8%. For comparison, the standard device based on ITO electrode with a structure of Glass/ITO/Al4083/PM6:Y6/PDINO/Ag demonstrates a *V*_OC_ of 0.83 V, *J*_SC_ of 26.2 mA/cm^2^, FF of 0.68 and a PCE of 14.8%. From the above data we can find that the ITO electrode-based device delivers similar *V*_OC_ and FF combined with higher *J*_SC_ than that of HC-PEDOT:PSS electrode-based device. The major difference should be caused by the higher conductivity of ITO than that of HC-PEDOT:PSS electrode. These results certify that the HC-PEDOT:PSS electrode could be used as an efficient transparent electrode for flexible solar cells and other similar devices.

## 3. Materials and Methods

### 3.1. Preparation of the Flexible Highly Conductivity PEDOT:PSS Electrode

The detailed preparation of HC-PEDOT:PSS electrode has been described as follows. Firstly, PES substrates (i-components) were adhered to glass substrates with polydimethylsiloxane (PDMS) sheets in between. Then the PH1000 aqueous solution (Heraeus, Hanau, Germany) was spin-coated onto the PES substrate which has been treated by plasma. Then the PES substrates coated with PH1000 (PES/PEDOT:PSS) films were peeled off from PDMS and dried at 150 °C for 10 min. The PES/PEDOT:PSS films were immersed in H_2_SO_4_ with different concentrations and at different temperatures (25–110 °C). The film was rinsed with deionized water three times. At last, the film was dried at 120 °C for 5 min. on a hot plate in air. The sheet resistance was measured by a four-point probe (RTS-8) and the film thickness measurement was performed using a surface profiler (Veeco Dektak 150). The conductivity was calculated based on the sheet resistance and the film thickness. The transmittance (*T*) and absorbance (A) of PES/PEDOT:PSS films were characterized by a UV-vis-NIR spectrophotometer (UV-3600, Shimadzu, Kyoto, Japan). Baseline correction was performed on air for the transmittance. A PANalytical X’Pert Pro multipurpose X-ray powder diffractometer (Cu Kα radiation) was used for XRD measurement. The temperature-dependent conductivity was measured under liquid N_2_ combined with heating conditions. Atomic force microscopy (AFM) images of the PEDOT:PSS films before and after H_2_SO_4_ treatment were taken using a Veeco NanoScope IV MultiMode in the tapping mode. Transmission electron microscope (TEM) measurement was performed by a type of LEO 912 OMEGA. The work function was characterized by Kelvin Probe (KP020).

### 3.2. Fabrication of Flexible All-Plastic Solar Cells

The flexible all-plastic solar cells with a device structure of PES/HC-PEDOT:PSS/PEI/P3HT:ICBA/EG-PEDOT:PSS were fabricated as follows. Firstly, PEI (Sigma-Aldrich, St. Louis, MO, USA) with a concentration of 0.2% was spin-coated onto the HC-PEDOT:PSS film at 5000 rpm for 1 min and annealed at 100 °C for 10 min to reduce the work function. The active layer was prepared by spin coating poly(3-hexylthiophene):indene-C60 bis-adduct (P3HT:ICBA, 1:1, weight ratio) with a total concentration of 40 mg/mL at 700 rpm for 40 s and solvent annealing at room temperature for 3 h. The thickness of P3HT:ICBA layer is about 200 nm. Finally, the PH1000 doped with 5wt% EG (Sigma-Aldrich) and 0.1wt% surfactant polyethylene glycol 2,5,8,11-tetramethyl-6-dodecyne-5,8-diolether (PEG-TmDD, TOYNOL Superwet-340, SurfyChem T&D Co., Ltd., Tianjin, China) which is used as the top electrode (denoted as EG-PEDOT:PSS) was prepared by transfer lamination technique, as previously described. Silver paint was applied onto HC-PEDOT:PSS and EG-PEDOT:PSS for electrical contact during the measurement. The cells were annealed in a N_2_-filled glovebox at 150 °C for 5 min to dry the PEDOT:PSS top electrode. Current density-voltage (*J-V*) characteristics were measured inside an N_2_-filled glovebox by using a sourcemeter (2400, Keithley Instruments, Cleveland, OH, USA) controlled by a LabVIEW program in the dark and under white light illumination (100 mW/cm^2^). The light intensity was calibrated using a silicon photodiode with KG5 filter (S1133, Hamamatsu).

### 3.3. Fabrication of ITO-Free OSCs

ITO-free solar cell devices were fabricated in the configuration of the traditional sandwich structure with Glass/HC-PEDOT:PSS/Al4083 as the positive electrode and a PDINO/Al negative electrode. The glass was pre-cleaned in an ultrasonic bath of detergent, deionized water, acetone, and isopropanol, and plasma-treated for 1 min. The electrode fabricated process was similar to PES/HC-PEDOT:PSS. A thin layer of PEDOT:PSS (Baytron PVP Al 4083) was prepared by spin-coating the PEDOT:PSS solution filtered through a 0.45 μm poly(tetrafluoroethylene) (PTFE) filter at 3000 rpm for 40 s on the Glass/HC-PEDOT:PSS electrode. Subsequently, PEDOT:PSS film was baked at 150 °C for 10 min in the air, and the thickness of the PEDOT:PSS layer was about 40 nm. The polymer PM6:Y6 (D:A = 1:1.2, 16 mg/mL in total) was dissolved in chloroform (CF) with the solvent additive of 1-chloronaphthalene (CN) (0.5%, *v*/*v*) and spin-cast at 3000 rpm for 30 s onto the PEDOT:PSS layer. The thickness of the photoactive layer is around 150 nm measured by Veeco Dektak 150. A bilayer cathode consisting of PDINO (~15 nm) capped with Ag (~90 nm) was thermally evaporated under a shadow mask with a base pressure of ca. 10^−4^ Pa. Finally, top electrodes were deposited in a vacuum onto the active layer. The active area of the device was about 5 mm^2^.

### 3.4. Fabrication of ITO-Based OSCs

ITO-based organic solar cell devices were fabricated in the configuration of the traditional sandwich structure with Glass/ITO/Al4083 as the positive electrode and a PDINO/Al negative electrode. The Glass/ITO electrode was pre-cleaned in an ultrasonic bath of detergent, deionized water, acetone, and isopropanol, and plasma-treated for 1 min. Then a thin layer of PEDOT:PSS (Baytron PVP Al 4083) was prepared by spin-coating the PEDOT:PSS solution filtered through a 0.45 μm poly(tetrafluoroethylene) (PTFE) filter at 3000 rpm for 40 s on the Glass/ITO electrode. Subsequently, PEDOT:PSS film was baked at 150 °C for 10 min in the air, and the thickness of the PEDOT:PSS layer was about 40 nm. The polymer PM6:Y6 (D:A = 1:1.2, 16 mg/mL in total) was dissolved in chloroform (CF) with the solvent additive of CN (0.5%, *v*/*v*) and spin-cast at 3000 rpm for 30 s onto the PEDOT:PSS layer. The thickness of the photoactive layer is around 150 nm measured by Veeco Dektak 150. A bilayer cathode consisting of PDINO (~15 nm) capped with Ag (~90 nm) was thermally evaporated under a shadow mask with a base pressure of ca. 10^−4^ Pa. Finally, top electrodes were deposited in a vacuum onto the active layer. The active area of the device was about 5 mm^2^.

## 4. Conclusions

In this work, a plastic electrode HC-PEDOT:PSS has been prepared by employing PEDOT:PSS and plastic substrate PES. This electrode treated with H_2_SO_4_ possesses a high conductivity of 2673 S/cm and high transmittance of over 90% at 550 nm. The superior conductivity mainly attributes to the removal of PSS and thus regular arrangement of PEDOT molecules. The binding force and effects between the PEDOT and PEI are investigated in detail, indicating a strong Coulomb force between these two molecules. All plastic solar cells with a device structure of PES/HC-PEDOT:PSS/PEI/P3HT:ICBA/EG-PEDOT:PSS demonstrate better performance than previous reports. ITO-free devices demonstrate a promising PCE of 12.8%. This research proves that HC-PEDOT:PSS is a promising electrode for flexible electronic devices and it is an effective method to enhance the device performance by boosting the conductivity of the electrode.

## Figures and Tables

**Table 1:** Statistics of conductivity and work function variations of PEDOT:PSS (PH1000) under different treating conditions.

Sample	Treating Method	Conductivity (S/cm)	Work Function (eV)
PEDOT:PSS (PH1000)	−	0.98	5.22
HC-PEDOT:PSS-1	80 wt% H_2_SO_4_	2673	5.12
HC-PEDOT:PSS (PEI)	0.2 wt% PEI	1744	4.22
HC-PEDOT:PSS-2	80 wt% H_2_SO_4_	2294	4.53

Note: The HC-PEDOT:PSS-1 represents the PEDOT:PSS film treated by 80 wt% H_2_SO_4_, the HC-PEDOT:PSS (PEI) represents the HC-PEDOT:PSS film treated by 0.2 wt% PEI solution, the HC-PEDOT:PSS-2 represents the HC-PEDOT:PSS (PEI) film treated by 80 wt% H_2_SO_4_.

**Table 2:** PCE statistics of single-junction all plastic solar cells, reported before and this work.

Bottom Electrodes	*V*_OC_ (V)	*J*_SC_ (mA/cm^2^)	FF	PCE (%)	References
HC-PEDOT:PSS	0.79 (0.79 ± 0.01)	8.99 (8.86 ± 0.97)	0.57 (0.57 ± 0.03)	4.05 (3.99 ± 0.41)	This work
EG-PEDOT:PSS	0.80	7.1	0.52	3.0	[24]
EG-PEDOT:PSS	0.80 ± 0.02	5.6 ± 0.5	0.55 ± 0.03	2.4 ± 0.2	[25]
P-PEDOT:PSS	0.84	6.60	0.60	3.3	[28]
EG-PEDOT:PSS	0.8	6.2	0.53	2.6	[34]
EG-PEDOT:PSS	1.55 ± 0.01	7.0 ± 1.0	0.59 ± 0.02	6.1 ± 0.4	

## Data Availability

The data that support the findings of this study are available from the corresponding author upon reasonable request.

## Supplementary Materials

The following supporting information can be downloaded at: https://www.mdpi.com/article/10.3390/molecules28062836/s1, Figure S1: Diffused surface reflectance spectra of PEDOT:PSS films treated by H_2_SO_4_ with different concentrations (0 wt%, 20 wt%, 40 wt%, 60 wt%, 80 wt%, 98 wt%).
