# Supplementary material for: High Conductivity, Semiconducting, and Metallic PEDOT:PSS Electrode for All-Plastic Solar Cells
Source: Molecules. 2023 Mar 21;28(6):2836. doi: 10.3390/molecules28062836 (PMC10059736; doi:10.3390/molecules28062836)
Supplement: Supplementary file 1 [file molecules-28-02836-s001.zip › molecules-2230819-supplementary.pdf]

High Conductivity, Semiconducting, and Metallic PEDOT:PSS Electrode for All-Plastic Solar Cells

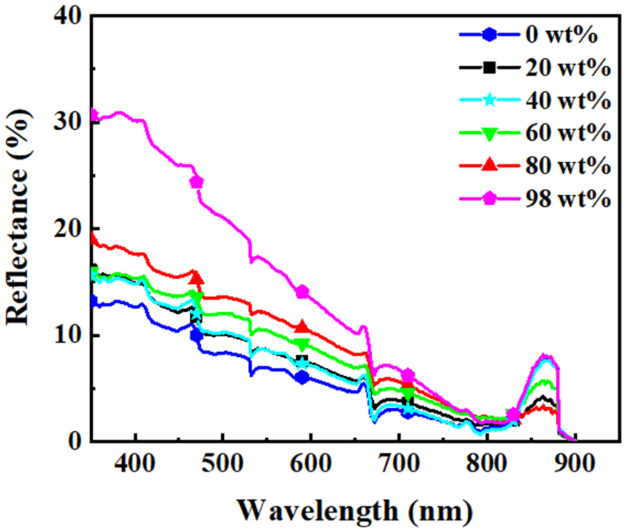

Figure S1. Diffused surface reflectance spectra of PEDOT:PSS films treated by H_2SO_4 with different concentrations (0 wt%, 20 wt%, 40 wt%, 60 wt%, 80 wt%, 98 wt%).
